# Low densities of immune cells indicate unfavourable overall survival in patients suffering from squamous cell carcinoma of the lung

**DOI:** 10.1038/s41598-024-64956-y

**Published:** 2024-06-20

**Authors:** Simone Steffens, Claudia Kayser, Anuschka Roesner, Justyna Rawluk, Severin Schmid, Eleni Gkika, Gian Kayser

**Affiliations:** 1 Institute of Pathology Naehrig Mattern Kayser, Bötzinger Strasse 60, Freiburg, Germany; 2 Institute of Surgical Pathology, University Medical Center Freiburg, Faculty of Medicine, University of Freiburg, Breisacher Strasse 115a, Freiburg, Germany; 3 Institute for Dermatopathology Laaf, Sasbacher Strasse 10, Freiburg, Germany; 4 Dental Clinic Zahnzentrum Roesner & Kollegen, Englerstraße 4a, Offenburg, Germany; 5 Department of Hematology and Oncology, University Medical Center Freiburg, Faculty of Medicine, University of Freiburg, Hugstetter Strasse 55, Freiburg, Germany; 6 Department of Thoracic Surgery, University Medical Center Freiburg, Faculty of Medicine, University of Freiburg, Hugstetter Strasse 55, Freiburg, Germany; 7 Department of Radiation Oncology, University Medical Center Bonn, Faculty of Medicine, University of Bonn, Venusberg-Campus 1, Bonn, Germany

**Keywords:** Oncology, Cancer, Cells, Lung cancer

## Abstract

Carcinogenesis and tumor proliferation are characterized by a complex interaction of cancer cells with the tumor microenvironment. In particular, a tumor-promoting effect can be assumed for the stroma and its fibroblasts. An influence of the immune system on non small cell lung cancer (NSCLC) is now also suspected. In our study, we examined 309 sections of squamous cell carcinoma (SCC), a subtype of NSCLC. We determined the cell densities and areas of the different tissues in SCC using the software QuPath. Spearman rank correlation showed a significant positive correlation between the different tumor cell densities and stromal cell densities, and between tumor cell densities and immune cell densities. Overall survival curves by the Kaplan–Meier method revealed a prominent negative curve in cases of low immune cell density. Based on our results, we can assume a positive influence of the tumor microenvironment, especially the stromal cells, on tumor proliferation in SCC. We have also revealed that low density of immune cells is prognostically unfavorable.

## Introduction

Lung cancer is one of the most prevalent malignant diseases worldwide. As most patients present in advanced stages and tumor progress will be unavoidable, lung cancer represents the number one cause of death among all cancers, worldwide^1^. For a long time squamous cell carcinoma (SCC) was the most prevalent subgroup of lung cancers. Since smoking habits have changed, lung adenocarcinomas have taken over this place. But still, indeed there is need to increase survival and therapeutic options for lung squamous cell carcinoma patients^2,3^.

The tumor microenvironment (TME) includes all non-malignant cells such as fibroblasts, immune or endothelial cells, as well as acellular components of the tumor. Many studies indicate an influence on carcinogenesis or tumor proliferation by the TME. Therefore, it represents a potential therapeutic target^4,5^. An essential part of the TME is the stroma, which plays an important role by structuring and remodeling the functional tissue. Futhermore, it hosts immune cells, responsible for anti-tumor, as well as tumor protective immune reactions.

An influence of the immune system on cancer is now undisputed, but current studies suggest a very complex role^6^. In non small cell lung cancer (NSCLC), these immune cell infiltrates include cells of both innate and adaptive immune defenses. Immune cells were found to be present in organized form in tertiary lymphoid structures (TLS). In addition to B-cell follicles containing proliferating germinal centers and a follicular dentritic cell network, TLS also exhibit T-cell clusters with mature dentritic cells. It is believed that in the TLS, the emergence of adaptive immunity is possible independent of secondary lymphoid organs. In lung tumors, the TLS can be referred to as tumor-induced bronchus-associated lymphoid tissue (Ti-BALT)^6,7^. A presence of Ti-BALT has been associated with favorable clinical outcome in several studies. In addition to these tumor-inhibiting properties of the immune system, other studies also suggest a tumor-promoting role. One possible explanation is the increased elimination of tumor variants with high immunogenicity, while tumors that can resist the immune response remain and proliferate^8,9^.

Upon this background, we have investigated the cellular composition with special focus on cellular densities in lung squamous cell carcinomas. The results of our study indicate a positive correlation between tumor and stromal cell densities in SCC and a prognostic impact of immune response on prolonged survival in SCC.

## Material and methods

### Cohort

**Figure 1:**
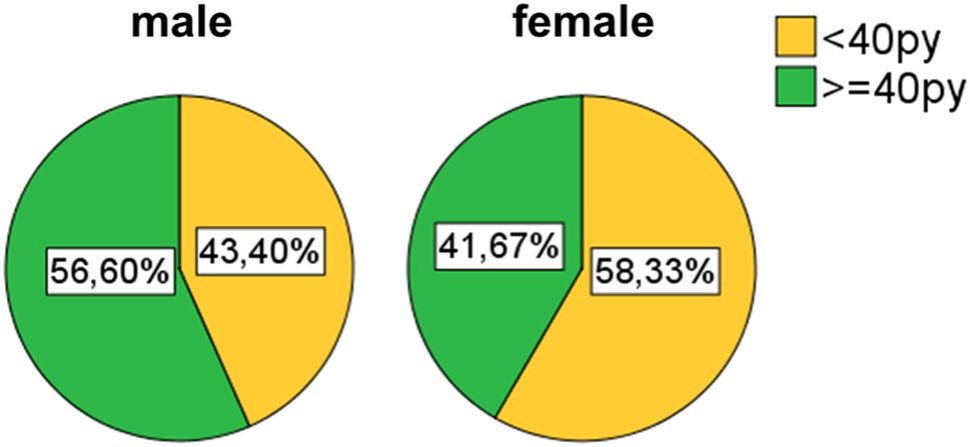
Age and gender distribution of cohort (163n).

**Figure 2:**
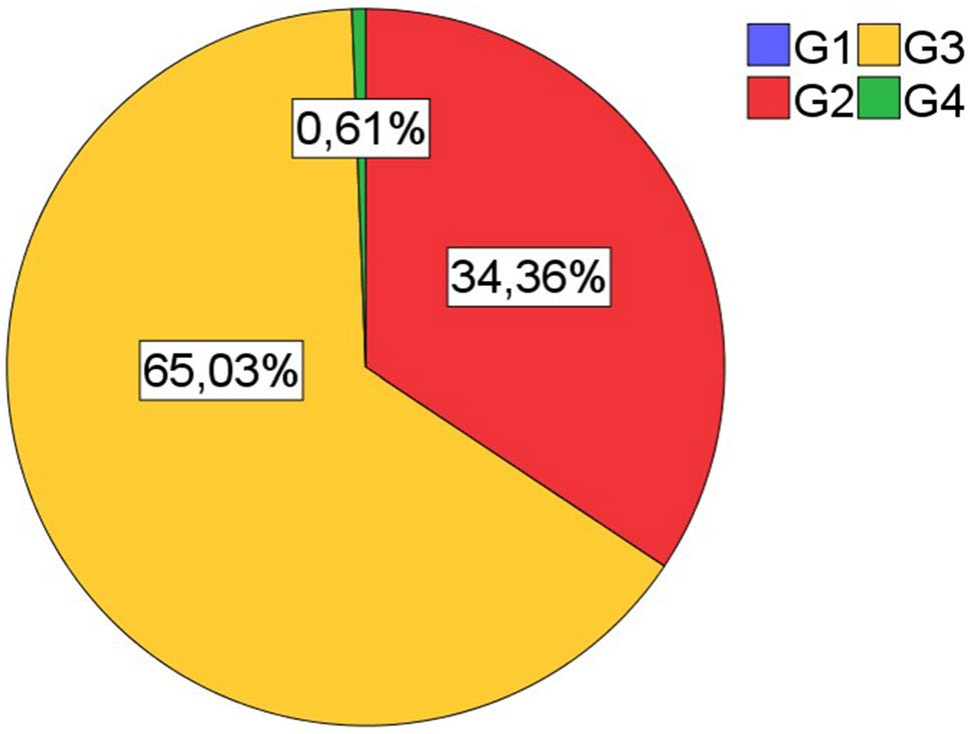
Distribution of grading in cohort (163n).

**Figure 3:**
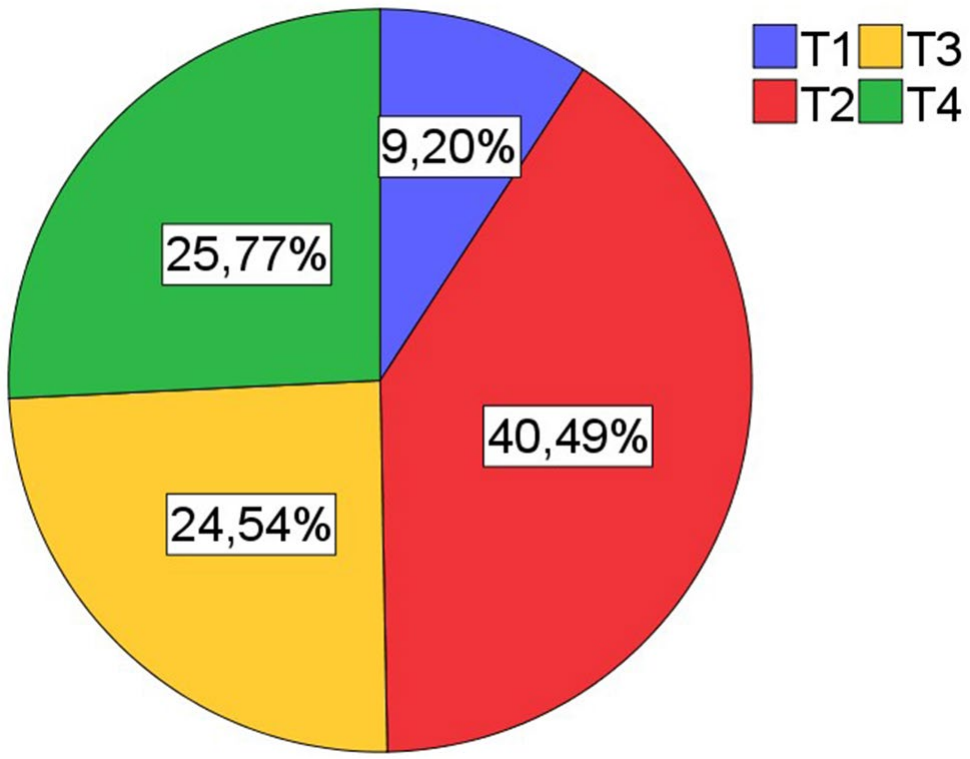
Distribution of primary tumor stage in cohort (163n).

**Figure 4:**
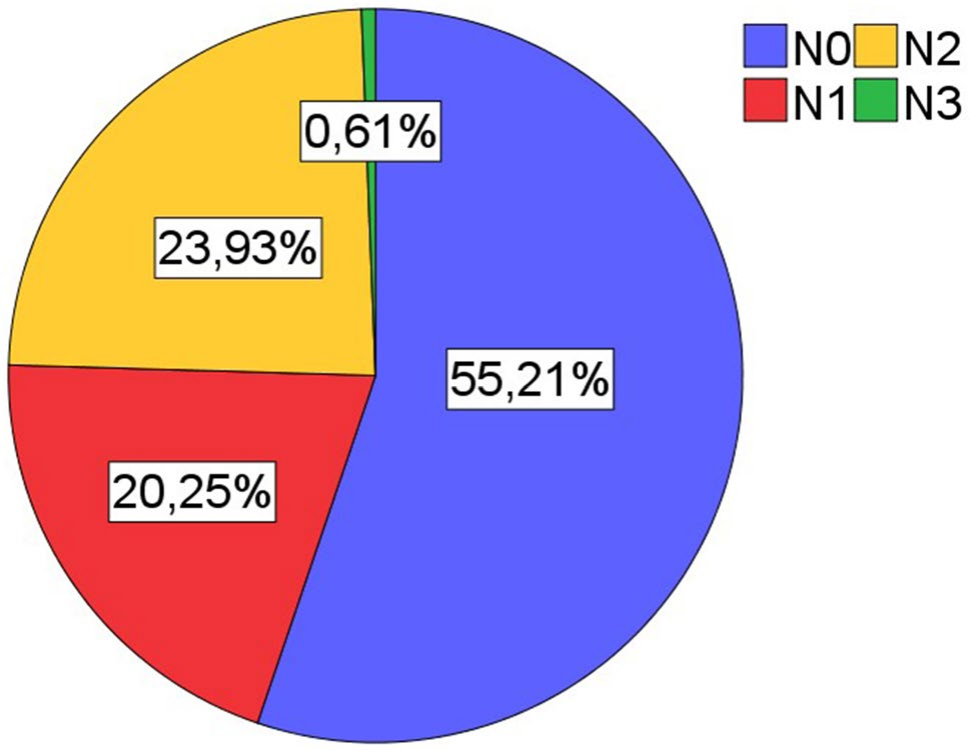
Distribution of lymph node stage in cohort (163n).

**Figure 5:**
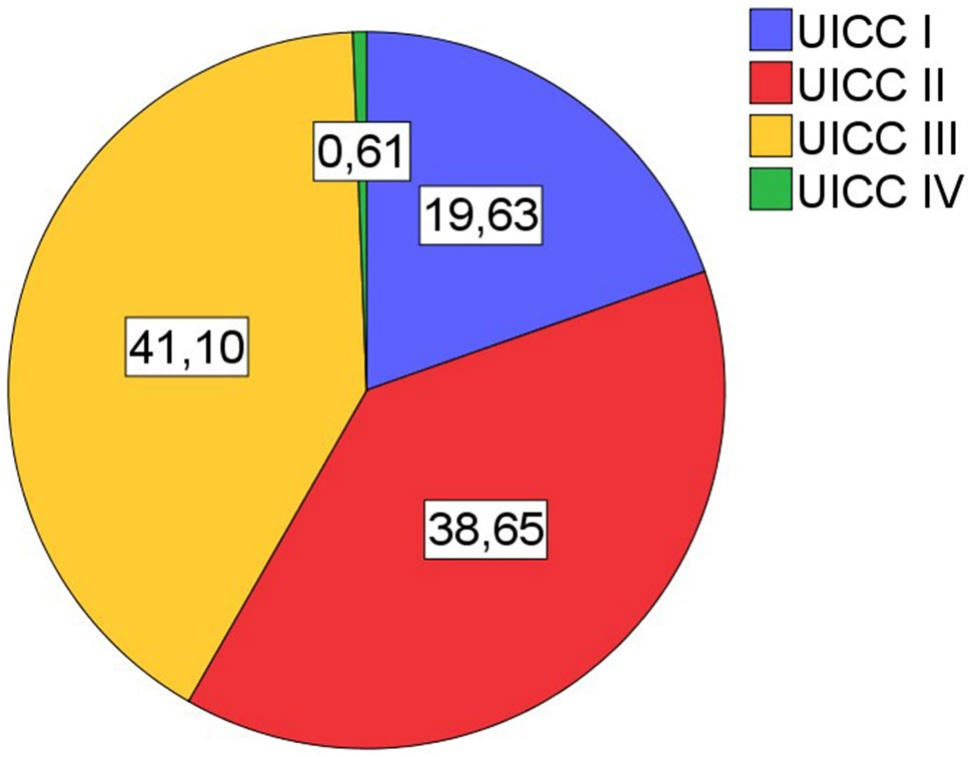
Distribution of UICC stage in cohort (163n).

Inclusion criteria for patients were primary lung carcinoma with histological type of squamous cell carinoma, operative treatment with curative intent, availability of sufficient tumor bearing paraffin blocks, availability of clinical data (especially follow up). All cases were retrieved in a timely sequentially manner from the archive of the Institute of surgical pathology, University Hospital Freiburg, depending on availability of sufficient formaline-fixed and paraffin embedded tissue. Patient selection was blinded with regard to lymphocytic tumor infiltrates. All histological specimens were reviewed with regard to diagnosis and tumor stage by board certified pathologists (GK, CK). For typing and staging of all cases the current WHO classification (2021) and the current UICC-TNM classification 8th edition (2017) were used. If necessary staging was adjusted according to the UICC-TNM classification 8th edition. The Tables 1, 2, and 3 and the Figs. 1, 2, 3, 4, and 5 give a summary of the clinical and pathological characteristics of our cohort.

**Table 1:** Age and gender distribution of cohort.

Value	Age (years)		
	Cohort (163n)	Men (137n)	Women (25n)
Mean	66	66	66
Median	67	67	69
Minimum	43	43	47
Maximum	81	81	77

**Table 2:** Grading and staging of cohort.

Value	Grading (163n)				Primary tumor (163n)				Lymph nodes (163n)				UICC Stage (163n)			
	G1	G2	G3	G4	T1	T2	T3	T4	N0	N1	N2	N3	I	II	III	IV
Frequency	0	56	106	1	15	66	40	42	90	33	39	1	32	63	67	1
Percent	0	34,4	65,0	0,6	9,2	40,5	24,5	25,8	55,2	20,2	23,9	0,6	19,6	38,7	41,1	0,6

**Table 3:** Overall survival.

Value	Overall survival (month)
Mean	52,7
Median	41
Minimum	27,7
Maximum	54,3

95% confidence intervals (CI).

### Tissue samples

165 patients were finally included in our study. The sections for analysis were selected without knowledge of clinical data. Preferred criteria for selection were a high proportion of tumor tissue and little healthy lung tissue, cartilage tissue, or lymph nodes. If available, three tumorbearing hematoxylin–eosin (HE) stained slides were selected for digitization, resulting in a total of 309 virtual slides to be analyzed.

### Digitization and analysis

**Figure 6:**
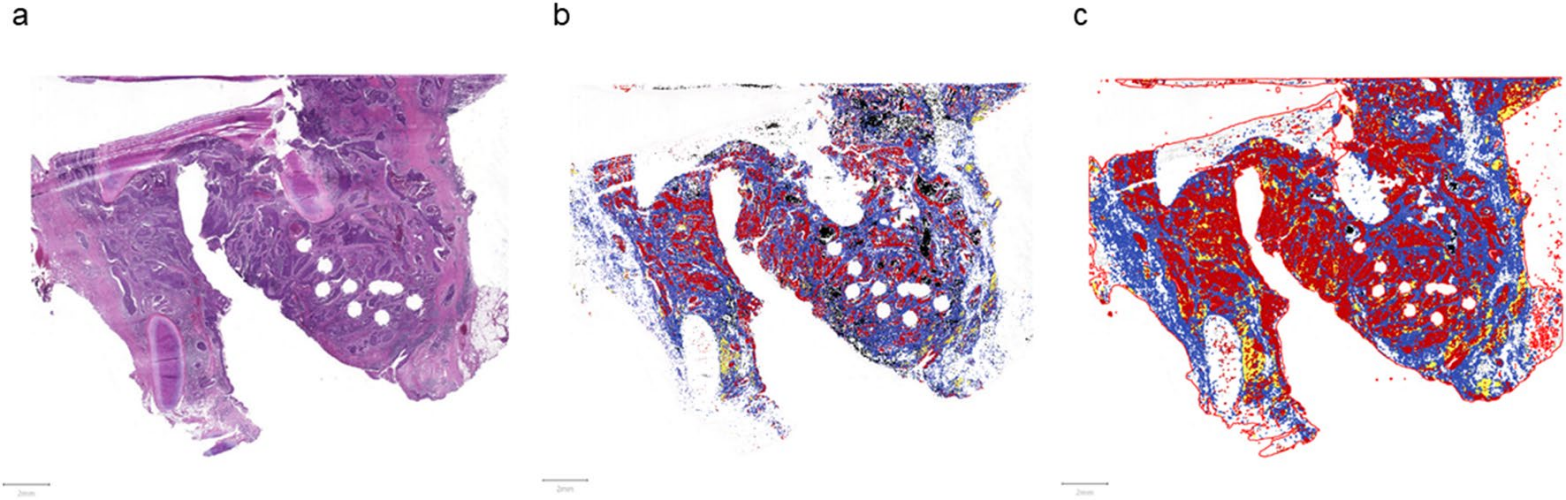
Classification of the tissue types—whole slide (**a**) unedited slide, (**b**) cells not filled, (**c**) cells filled. red: tumor, blue: stroma, yellow: immune cells, black: necrosis, white: other tissue (excluded).

**Figure 7:**
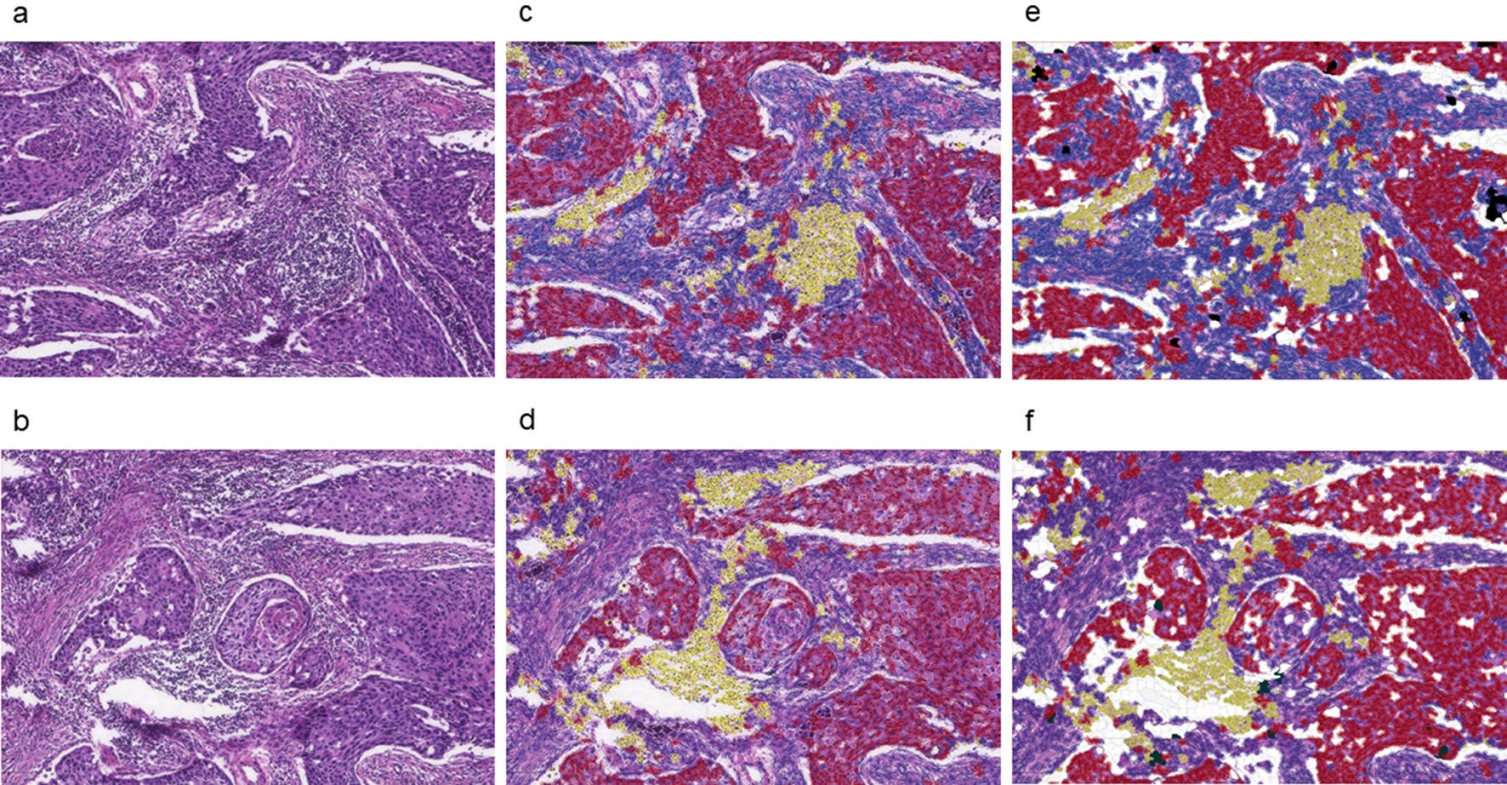
Classification of the tissue types—image detail (**a**) + (**b**) unedited slide, (**c**) + (**d**) cells not filled, (**e**) + (**f**) cells filled. red: tumor, blue: stroma, yellow: immune cells,black: necrosis, white: other tissue (excluded).

The selected slides were digitized using a whole slide scanner (Pannoramic SCAN, 3DHISTECH, Budapest, Hungary; 20 × objective, NA 0.8, pixel-resolution 0.22 µm^2^). For image processing and analysis, we used the open source software Quantitative Pathology (QuPath) (Version 0.1.2, Queen’s University, Belfast, Nordirland, https://qupath.readthedocs.io/en/stable/). This software provides tools to classify tissue types, count cells or calculate areas, based on Deep Learning approaches^10^. Using selected tools, we were able to classify the tissue types tumor, stroma, immune cells and necrosis (Figs. 6 and 7). Tumor stroma is defined as the non-epithelial part of carcinomas. Since vascular cells are usually only a very minor part in tumor stroma with respect to cellularity (not to be confused with percentage of area) we focused of spindle stromal cells, i.e. fibroblasts and fibrocyts. In concordance with internationally accepted and used scoring systems (e.g. CPS, IC-score), in the term immune cells were all subtypes cellular classes included which are involved in immune reactions, i.e. lymphocytes and macrophages.

Thus, digital slides were subdivided using QuPath into areas with (1) tumor tissue with (1a) epithelial tumor compartment and (1b) tumor-stroma compartment, (2) normal tissue and (3) immune cell compartment, i.e. denselymphocyte aggregates without tertiary lymphocytic architecture. Within these tissue classes QuPath calculated cell densities, accordingly, additionally we analysized the immune cell densitiy with regard to the whole tumor bearing slide. Distinction between immune cells, i.e. lymphocyts and macrophages, and stromal cells, i.e. fibroblasts and fibrocyts, was done on hematoxylin–eosin stained slides upon their morphology. Existing lymph nodes were excluded. To obtain reliable results, all sections were screened after automated classification. If the tissue classification was inadequate, the sections were reclassified. The respective area sizes were determined and the cells were counted.

### Data and variables

The results of the individual sections were added per case and the mean value was calculated. The values were then merged with the patient data. The values determined in QuPath only concerned cell number (tumor, stromal and immune cells) and area size (whole slide, tumor tissue, stroma, immune cell tissue and necrosis). In order to be able to investigate further parameters, additional variables were created (Table 4). Two cell densities were determined for each cell class, one per area of the respective class and one per area of the overall tumor or whole slide. The overall tumor was defined as sum of the area size for tumor tissue, stroma and necrosis.

**Table 4:** Variables.

Name of variable	Calculation of variable
Density of tumor cells (mm^2^)	Number of tumor cells/area of tumor tissue × 1.000.000
Tumor cell density/overall cellular density (mm^2^)	Number of tumor cells/area of overall tumor × 1.000.000
Density of stromal cells (mm^2^)	Number of stromal cells/area of stroma × 1.000.000
Stromal cell density/overall cellular density (mm^2^)	Number of stromal cells/area of overall tumor × 1.000.000
Density of immune cells (mm^2^)	Number of immune cells/area of immune tissue × 1.000.000
Immune cell density/virtual slide area (mm^2^)	Number of immune cells/area of the whole slide × 1.000.000

The area of the overall tumor is the sum of the area size of tumor tissue, stroma and necrosis.

### Statistical analysis

Normal distribution of cell densities was tested using the Kolmogorov Smirnov test. The correlation of nominal, ordinal or numerical data were examined. For normal distributed variables we used one sample t-test or ANOVA and the Pearson correlation. Non-parametric data was analyzed with Wilcoxon rank-sum test or Kruskal–Wallis test and Spearman rank correlation. Correlation analysis was performed for 163 cases. Overall survival curves were determined using the Kaplan–Meier method. Cutoff for overall survival was 120 months and the respective quartiles of the variables were compared. By means of the log-rank test, the overall survival of the quartiles could additionally be tested for significant differences. Overall survival could only be calculated in 157 cases due to lack of clinical data. The median survival time was also determined and reported with the 95% confidence interval (CI). Statistical analyses was performed using SPSS software (Version 21, International Business Machines Corporation, Armonk, USA). A *p*-value less than 0.05 was considered statistically significant.

### Ethical approval

The study was approved by the institutional ethics committee (EK 10/12) and was conducted in accordance with the ethical principles stated in the most recent version of the declaration of Helsinki. According to this approval, Individual written informed consent of the included patients has been waived, due to the use of material which had been archived for at least 3 years by the ethics commettee (Ethik-Kommission (ethics committee), Albert-Ludwigs-University Freiburg, project number: EK10/12).

## Results

### Correlation between different cellular densities

**Figure 8:**
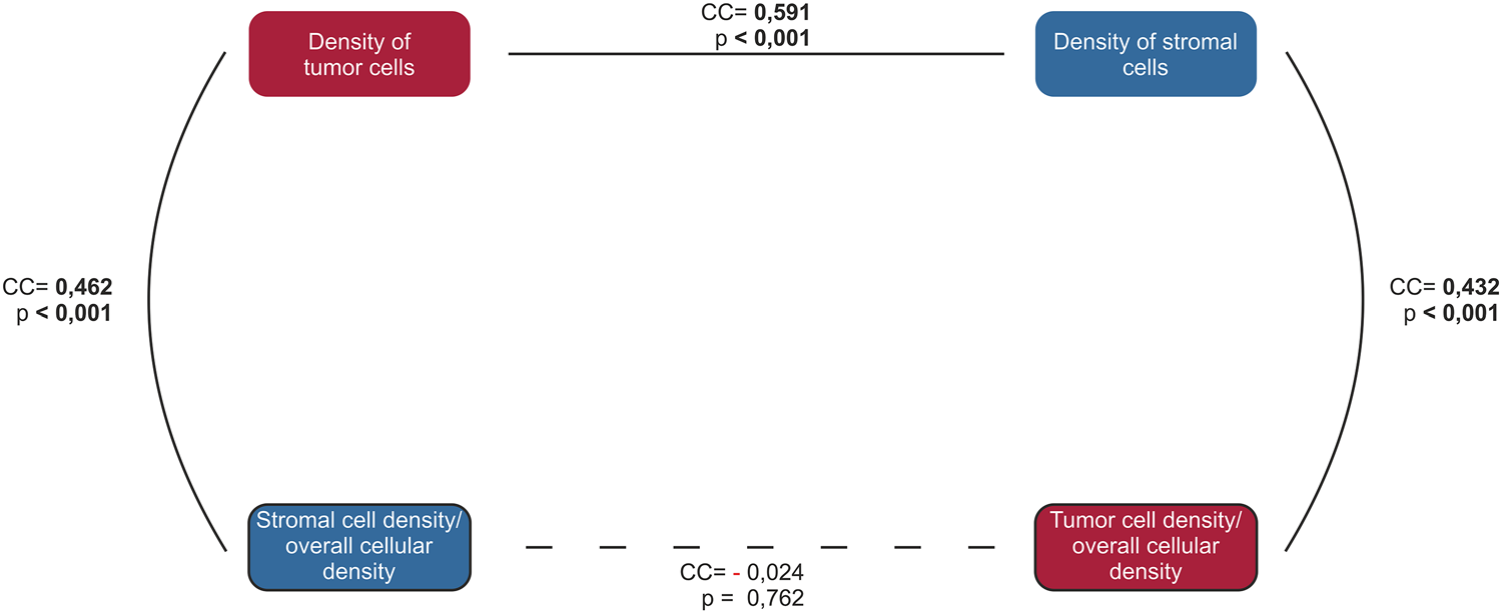
Overview of the correlations between “density of tumor cells” and “density of stromal cells”, cc: correlation coeffizient.

**Figure 9:**
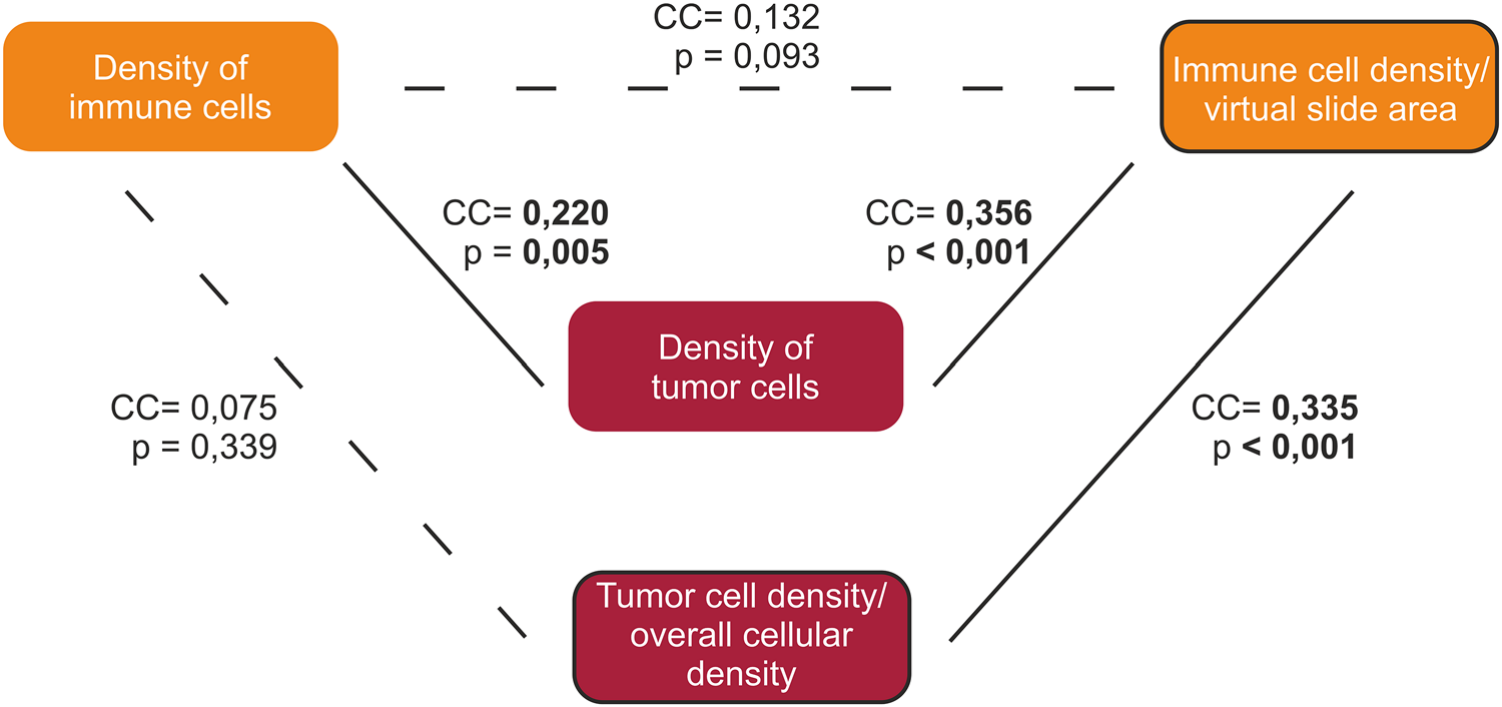
Overview of the correlations between “density of tumor cells” and “density of immune cells”, cc: correlation coeffizient.

We found significant positive correlations (*p* < 0.001) between “density of tumor cells” and “tumor cell density/overall cellular density”, as well as “density of stromal cells ” and “stromal cell density/overall cellular density ”, respectively. (Table 5). Likewise, “density of tumor cells” correlated significantly positively with all other cell densities examined. However, the various correlation coefficients differed markedly. In terms of “tumor cell density/overall cellular density ”, there was a significant positive correlation (*p* < 0001) with “immune cell density/virtual slide area” and “density of stromal cells”, the correlation coefficients were approximately equal (Table 6, Figs. 8 and 9).

**Table 5:** Correlation within a cell class (163 cases).

Variable 1	Variable 2	Correlation	*p*-value
Density of tumor cells*	Tumor cell density/overall cellular density	0.485	**< 0.001**
Density of stromal cells**	Stromal cell density/overall cellular density	0.605	**< 0.001**
Density of immune cells*	Immune cell density/virtual slide area	0.132	0.093

*Spearman rank correlation.

**Pearson correlation.

Significant values are in bold.

**Table 6:** Correlation of tumor cell density (163 cases).

Variable 1	Variable 2	Correlation	*p*-value
Density of tumor cells*	Density of immune cells	0.220	**0.005**
	Immune cell density/virtual slide area	0.356	**< 0.001**
	Density of stromal cells	0.591	**< 0.001**
	Stromal cell density/overall cellular density	0.462	**< 0.001**
Tumor cell density/overall cellular density*	Density of immune cells	0.075	0.339
	Immune cell density/virtual slide area	0.335	**< 0.001**
	Density of stromal cells	0.432	**< 0.001**
	Stromal cell density/overall cellular density	− 0.024	0.762
Density of stromal cells*	Density of immune cells	0.206	**0.008**
	Immune cell density/virtual slide area	0.411	**< 0.001**
Stromal cell density/overall cellular density*	Density of immune cells	0.493	**< 0.001**
	Immune cell density/virtual slide area	0.205	**0.009**

*Spearman rank correlation.

**Pearson correlation.

Significant values are in bold.

### Survival analysis for the “density of immune cells”

**Figure 10:**
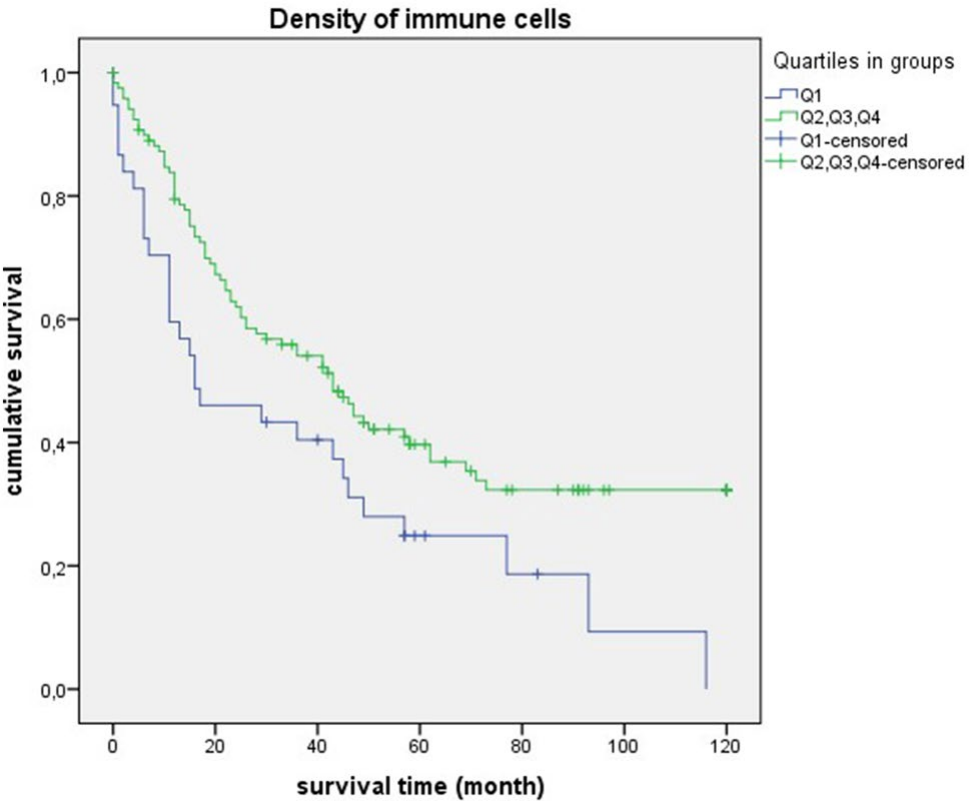
Survival curves for “density of immune cells” Survival time: 0–120 month, Number of cases Q1: 38. Number of cases Q2 + Q3 + Q4: 119, *p*-value: 0.010, Median: 16 month (Q1), 95% CI 0.00–35.04, 43 month (Q2 + Q3 + Q4), 95% CI 31.89–54.10.

**Figure 11:**
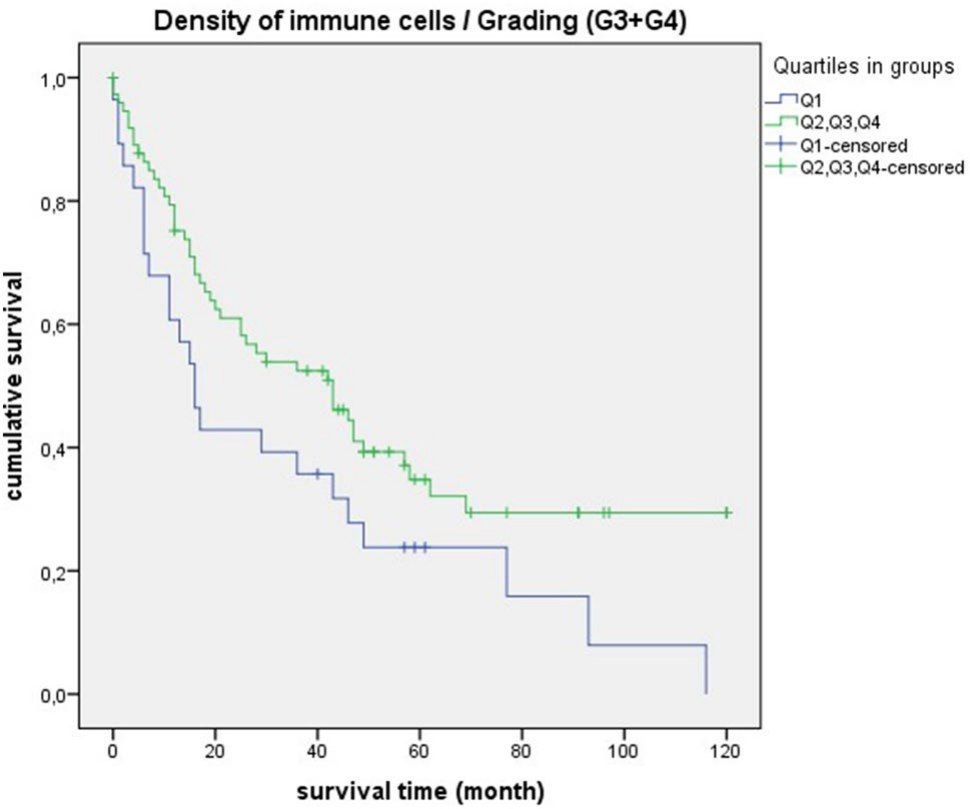
Survival curves for “density of immune cells” with poorly differentiated tumors. Survival time: 0–120 month, Number of cases Q1: 28, Number of cases Q2 + Q3 + Q4: 74, *p*-value: 0.035, Median: 16 month (Q1), 95% CI 10.82–21.17, 43 month (Q2 + Q3 + Q4), 95% CI 23.59–62.40.

**Figure 12:**
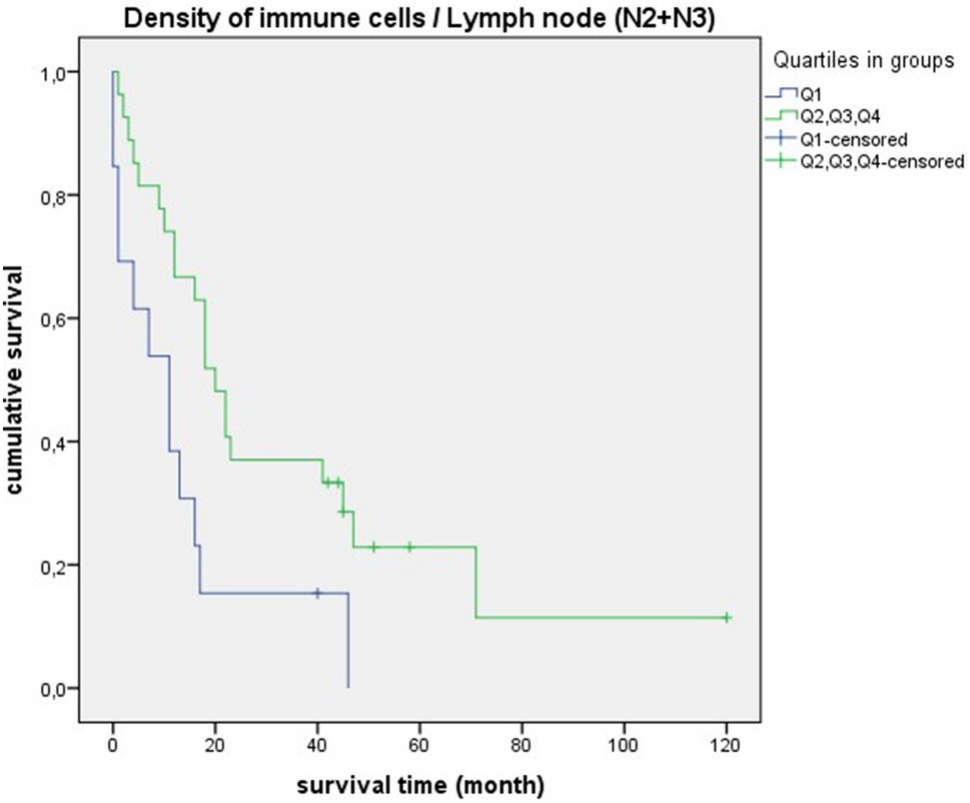
Survival curves for “density of immune cells” with mediastinal lymph node metastases. Survival time: 0–120 month, Number of cases Q1: 13, Number of cases Q2 + Q3 + Q4: 27, *p*-value: 0.012, Median: 11 month (Q1), 95% CI 2.97–19.02, 20 month (Q2 + Q3 + Q4), 95% CI 14.91–25.08.

**Figure 13:**
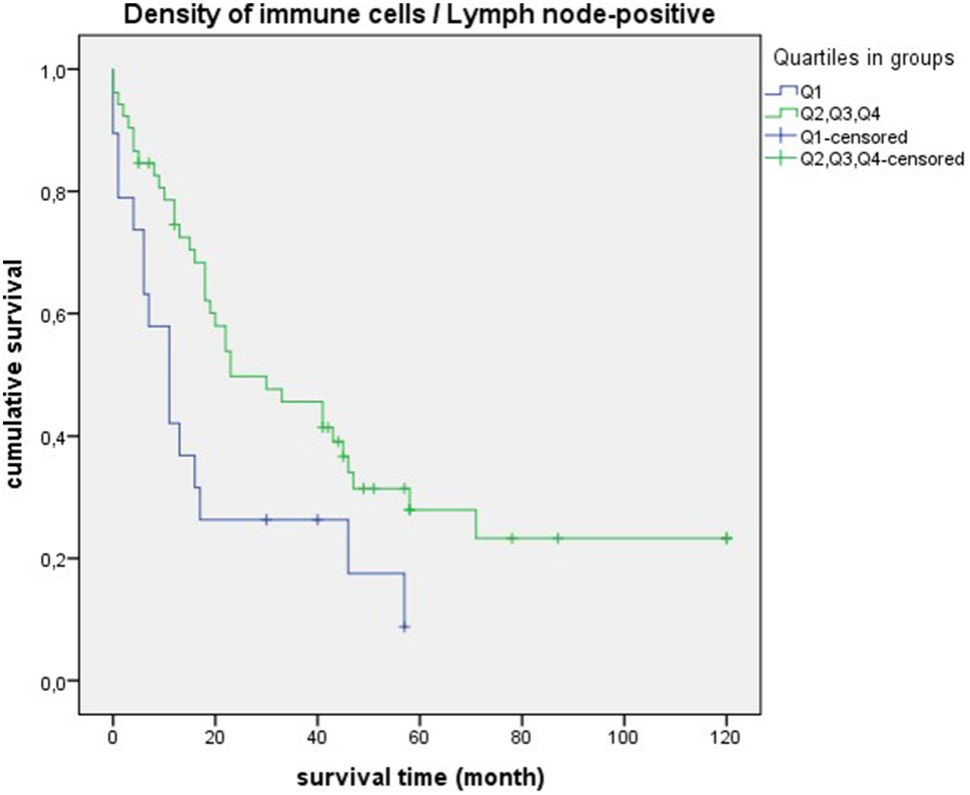
Survival curves for “density of immune cells” in lymph node-positive cases (N1 + N2 + N3). Survival time: 0–120 month, Number of cases Q1: 19, Number of cases Q2 + Q3 + Q4: 52, *p*-value: **0.016,** Median: 11 month (Q1), 95% CI 5.37–16.62, 23 month (Q2 + Q3 + Q4), 95% CI 5.27–40.72.

**Figure 14:**
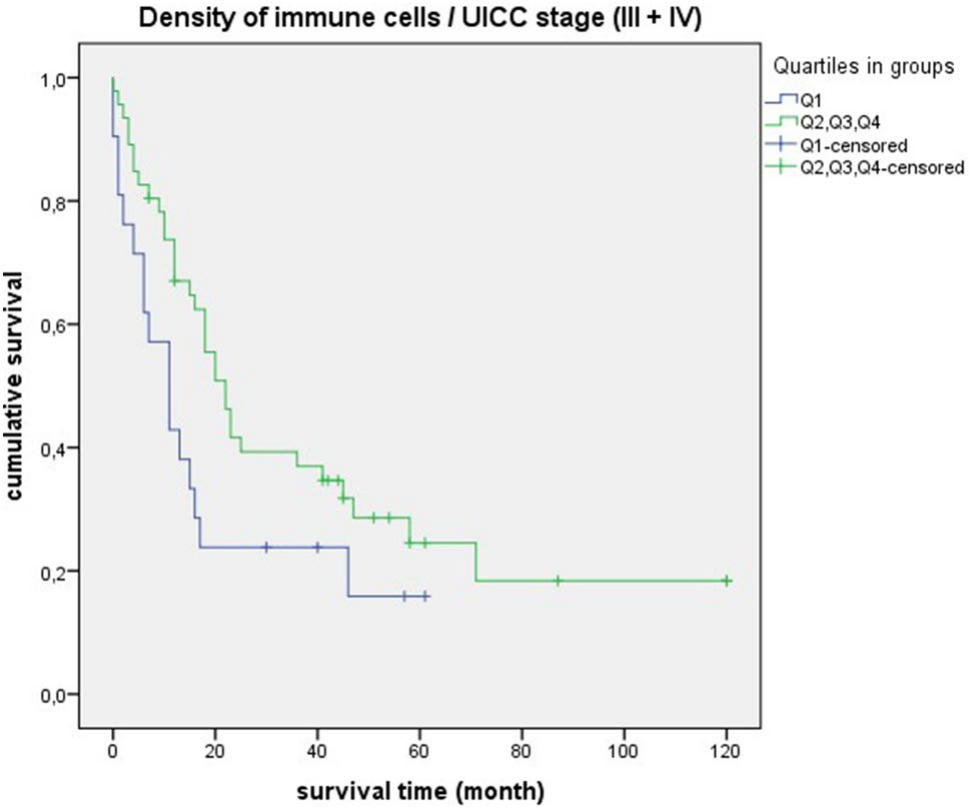
Survival curves for “density of immune cells” in UICC stage III + IV. Survival time: 0–120 month, Number of cases Q1: 21, Number of cases Q2 + Q3 + Q4: 46, *p*-value: 0.056, Median: 11 month (Q1), 95% CI 5.07–16.92, 22 month (Q2 + Q3 + Q4), 95% CI 16.69–27.30.

In the survival statistics of “density of immune cells”, the negative curve of quartile 1 was particularly prominent. Compared to the cases in quartiles 2, 3 and 4, survival was significantly shorter (quartil 1: median = 16 month, 95% CI 0.00–35.04; quartil 2, 3, 4: median = 43 month, 95% CI 31.89–54.10; *p* = 0.010) (Fig. 10). Similar significant results could be found in relation to different grading and staging parameters. There was a significant overall survival disadvantage for quartile 1 in relation to poorly differentiated tumors (G3 + G4) (quartil 1: median = 16 month, 95% CI 10.82–21.17; quartil 2, 3, 4: median = 43 month, 95% CI 23.59–62.40; *p* = 0.035) (Fig. 11). In connection with mediastinal lymph node metastases (N2 + N3), there was also unfavourable overall survival in quartile 1 (quartil 1: median = 11 month, 95% CI 2.97–19.02; quartil 2, 3, 4: median = 20 month, 95% CI 14.91–25.08; *p* = 0.012) (Fig. 12). A comparable result could be determined for lymph node-positive cases (pN +) (quartil 1: median = 11 month, 95% CI 5.37–16.62; quartil 2, 3, 4: median = 23 month, 95% CI 5.27–40.72; *p* = 0.016) (Fig. 13). In terms of the UICC stage III and IV the survival statistic also showed a poorer survival in quartile 1 compared to the quartiles 2, 3 and 4. However, the *p*-value was just above the statistical threshold (quartil 1: median = 11 month, 95% CI 5.07–16.92; quartil 2, 3, 4: median = 22 month, 95% CI 16.69–27.30; *p* = 0.056) (Fig. 14).

It is striking that the survival curves of cases with poorly differentiated tumors (G3 + G4) are very similar to the curves of “densitiy of immune cells” in general. This is also reflected by equal median values (Fig. 10 and 11). For quartile 1, the median survival time is 16 months, for quartiles 2 + 3 + 4 it is much higher at 43 months. For cases with lymph node involvement (N2 + N3/lymph node-positive) or in UICC stadium III + IV, the median values are also close (Figs. 12, 13, and 14). For quartile 1, the survival time is 11 months respectively. For quartiles 2 + 3 + 4, the median is between 20 to 23 months. Therefore, the median survival for quartiles 2 + 3 + 4 was longer for cases with poorly differentiated tumors (G3 + G4) than for the staging parameters (N2 + N3/lymph node-positive/UICC stage III + IV).

## Discussion

The aim of our study was to use QuPath to provide a high quality classification of the different tissue types and to determine the respective cell number and area size. Because there is high variability in an evaluation such as cell recognition by pathologists, digital pathology could be of use in these areas to support diagnostic pathologists^11,12^. Correlation analysis revealed significant positive correlations between “density of tumor cells” and “tumor cell density/overall cellular density” and between “density of stromal cells” and “stromal cell density/overall cellular density”. Only in the case of rectified correlation within a cell class can a correct classification be assumed. The high significance thus illustrates reliable cell detection and classification of tumor and stromal cells. The study situation on the quality and reliability when using automated classification of tissue types with QuPath software is very scarce. Therefore, validation of our methodology based on the results of correlation analysis was essential.

It is well known that beside genetic and cellular factors, the stroma plays an essential role in carcinogenesis, tumor proliferation and metastasis. However, the relationships and interactions between tumor tissue and stroma are not yet fully understood and different study results indicate the need for further research^5^.

The influence of tumor cells on the surrounding stroma through expression of growth factors and a resulting accelerated fibroblast proliferation or angiogenesis has been known for a long time^13,14^. However, several studies have now also demonstrated a modulatory effect of stromal cells on carcinogenesis in adjacent epithelia^15^. Bhowmick et al. showed in a mouse model that inactivation of transforming growth factor beta (TGF-β) receptor in cancer associated fibroblasts (CAFs) leads to epithelial proliferation, which may even result in invasive carcinoma in selected tissues^15^. With regard to NSCLC, no reliable marker as a clinically relevant predictor for tumor proliferation has been found so far. According to current studies, however, CAFs are also assumed to play an important role^16^.

Our analysis showed a significant positive correlation of the “density of tumor cells” with the “density of stromal cells ” and the “stromal cell density/overall cellular density”. Also between “tumor cell density/overall cellular density” and “density of stromal cells” (Fig. 8). Thus, the results clearly support a positive correlation between tumor and stromal cell densities in SCC. It should be noted that our study refers mostly to SCC at UICC stage II and III (Table 2). In the literature, the role of stroma in early-stage cancer is increasingly described as tumor-suppressive, whereas the tumor-promoting influence is suspected only after a certain aggressiveness of the tumor^5^. In this context, it is clear that the stromal approach to cancer therapy is currently considered promising and understanding the biological mechanisms of action between tumor tissue and stroma is imperative^17^.

The immune response plays an important role in the surveillance and control of tumors. Especially with regard to new therapy options, the immune system has therefore increasingly become in focus of cancer research within recent years^6^. Although elimination of tumors is not always possible solely through the immune reaction, a tumor-inhibiting effect has been observed. However, it has also been found that the immune system may partially facilitate tumor progression^18^.

Since distinction between preexisting lymphatic tissue and secondary immune reaction leading to tertiary lymphatic structures is not possible and on the other hand, dens lymphocytic infiltrates without tertiary lymphatic architecture can most probably accounted for an immune reaction induced by the tumor itself, we focused our analytic approach to dense immune aggregates as definition of “immune cell compartment” and excluded tertiary lymphatic structures.

Our results indicate a positive correlation between tumor cell density and immune cell density (Fig. 9). Since a tumor-inhibiting role of the immune system is expected, the positive correlation seems paradoxical at first. There are similar findings for NSCLC and other cancer entities^8,19^. Velcheti et al. demonstrated an association of programmed death ligand 1 (PD-L1) expression with increased lymphocytic infiltrates in NSCLC^19^. In addition, tumor-promoting and immunosuppressive effects have been reported in specifically activated macrophages or regulatory T cells^8^. Recently, preclinical studies have demonstrated increased secretion of immunosuppressive enzymes in tumor-infiltrating dendritic cells (DCs) and linked it to accelerated tumor growth^20,21^. The positive correlation we found between tumor cell density and immune cell density could be attributed to such mechanisms.

Several studies also provided evidence for a prognostic significance of the immune response. In a study, including 219 patients with lobectomy for stage I NSCLC, a higher proportion of tumor-infiltrating lymphocytes was shown to correlate with a lower risk of disease recurrence and improved disease-free survival^22^. Our results of survival analysis for “density of immune cells” showed an unfavorable prognosis with low immune cell density (Quartil 1). Accordingly, we confirmed the prognostic impact of immune response on prolonged survival in SCC.

Dieu-Nosjean et al. demonstrated in a retrospective study that poor clinical outcome directly correlated with low number of TLS^7^. Goc et al. also found poor survival for NSCLC patients with few TLS^6,23^. These results support our hypothesis of a prognostically important role for an organized immune response.

In survival analysis of “density of immune cells”, shorter survival was consistently seen in quartile 1. Significant values were obtained in poorly differentiated tumors (G3 + G4), mediastinal lymph node metastases (N2 + N3) and lymph node-positive cases. A statistical trend was present in UICC stage III + IV. Thus, we demonstrated for SCC that a low density of immune cells is prognostically unfavorable. The clinical relevance of a qualitative immune cell response is again highlighted by these results. In a comparable retrospective study, a positive impact on prognosis was demonstrated by a dense infiltration of CD4 + T cells and CD8 + T cells in the tumor stroma of NSCLC^24^. In relation to colorectal cancer, it has already been demonstrated that patients with high immune levels have a longer overall survival. Remarkably, in this study, a proposed immune score was also found to be a better prognostic factor than other clinical parameters such as TNM staging^25^.

## Conclusion/clinical relevance

In our study we found a positive correlation between tumor and stromal cell densities in SCC of the lung and conclude that there is a tumor-promoting influence after a certain aggressiveness of the tumor. The demonstrated positive correlation between tumor cell density and immune cell density supports the hypothesis of various studies^8,19^. There is therefore much evidence that immune cell density can have a tumor-promoting effect. In conclusion, we are able to demonstrate that immune cell infiltrates and immune cell density are important for clinical outcome in lung SCC.

## Data Availability

The dataset used and analysed during the current study are available from the corresponding author on reasonable request.
